# Supplementary material for: HSV-1 Infection of Epithelial Dendritic Cells Is a Critical Strategy for Interfering with Antiviral Immunity
Source: Viruses. 2022 May 14;14(5):1046. doi: 10.3390/v14051046 (PMC9147763; doi:10.3390/v14051046)
Supplement: Supplementary file 1 [file viruses-14-01046-s001.zip › viruses-1677478-supplementary.pdf]

Supplementary

HSV-1 infection of epithelial dendritic cells is a critical strategy for interfering with antiviral immunity

Yang Gao*, Jishuai Cheng*, Xingli Xu, Xueqi Li, Jingjing Zhang, Danjing Ma, Guorun Jiang, Yun Liao, Shengtao Fan, Zhenye Niu, Rong Yue, Penglan Chang, Fengyuan Zeng, Suqin Duan, Ziyan Meng, Xiangxiong Xu, Xinghang Li, Dandan Li, Li Yu, Lifen Ping, Heng Zhao, Mingtian Guo, Lichun Wang, Yafang Wang, Ying Zhang†, Qihan Li†

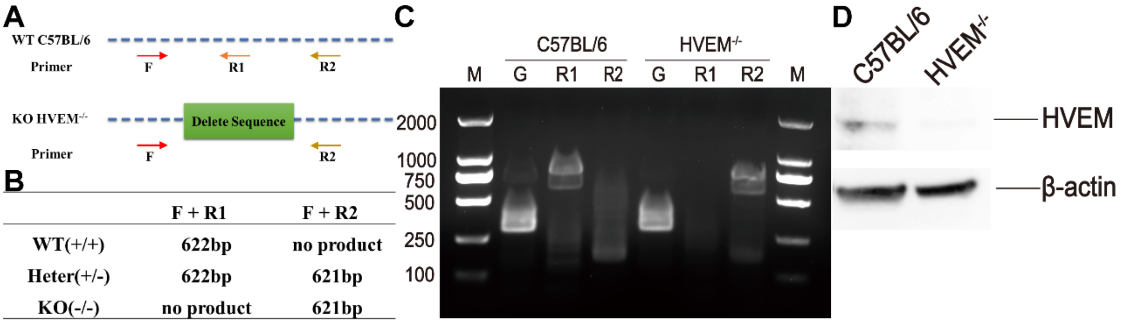

Supplementary Figure S1. Analysis of HVEM gene deficiency by nucleic acid electrophoresis and western blot. **(A)** Principles of primer design for knockout identification. **(B)** Criteria with PCR for Gene Knockout in Mice. **(C)** Nucleic acid gel electrophoresis for mice genotype identification. C57BL/6 indicates WT mice, HVEM$^{-/-}$ indicates C57BL/6 mice with HVEM gene knockout. M stands for marker, G stands for GAPDH. The presence of R1 and the absence of R2 stands for WT, the absence of R1 and the presence of R2 stands for knockout. **(D)** Criteria with Western blot for Gene Knockout in Mice. C57BL/6 indicates WT mice, HVEM$^{-/-}$ indicates C57BL/6 mice with HVEM gene knockout. β-actin as internal reference.

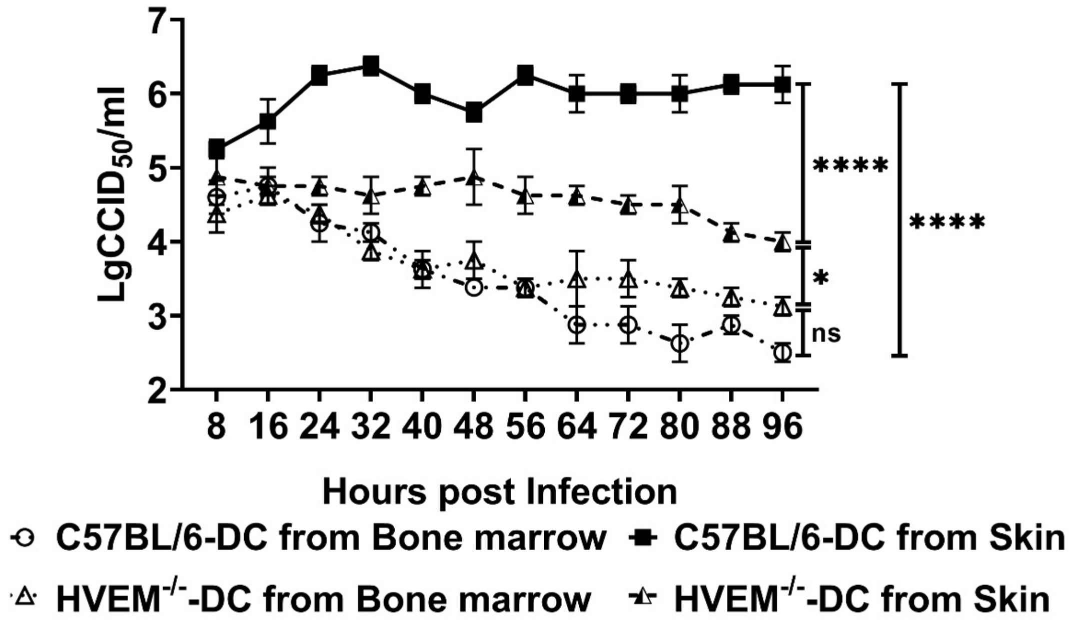

Supplementary Figure S2. Analysis of the viral titer in dendritic cells from the skin and bone marrow of C57BL/6 and HVEM$^{-/-}$ mice infected with HSV-1 and cultured in vitro at different time points. (n=3 per timepoint in each group). Statistical significance was assessed by two-way ANOVA with Holm–Sidak adjustment for multiple comparisons (*, $p<0.05$; ****, $p< 0.0001$; ns, no significant difference).

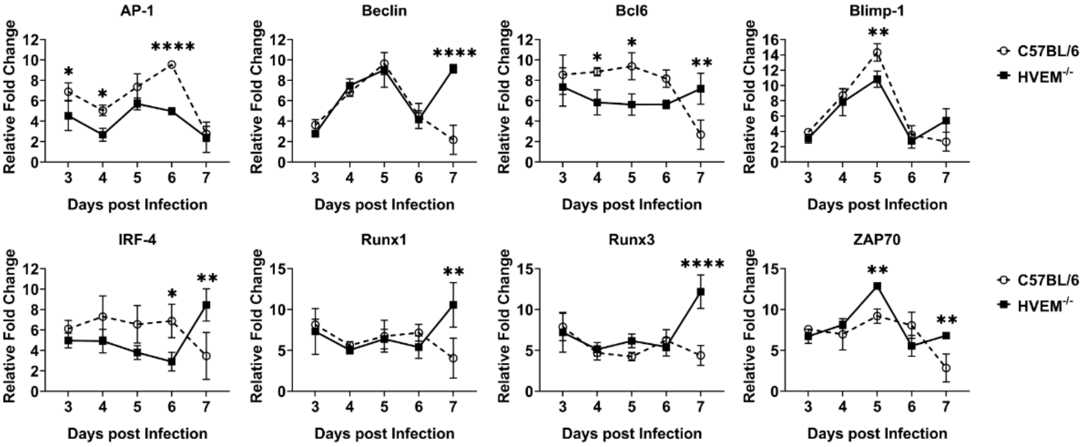

Supplementary Figure S3. Analysis of the transcription levels of various signaling molecules and transcription factors related to T cell proliferation in the lymph nodes. (n=3 per time point in each group). Statistical significance was assessed by two-way ANOVA with Holm–Sidak adjustment for multiple comparisons (*, p<0.05; **, p<0.01; ****, p< 0.0001).

Supplementary Table S1. Primers used for quantitative RT-PCR in the study.

Primer name	Sequence (5'–3')
AP-1-F	CCTTCTACGACGATGCCCTC
AP-1-R	GGTTCAAGGTCATGCTCTGTTT
Bcl6-F	CCGGCACGCTAGTGATGTT
Bcl6-R	TGTCTTATGGGCTCTAAACTGCT
Beclin-F	GCTGGCCTTGGAGGAGGAGAG
Beclin-R	CCGCCTCAGCCTGGACCTTC
Blimp-1-F	TTCTCTTGGAAAAACGTGTGGG
Blimp-1-R	GGAGCCGGAGCTAGACTTG
CCL28-F	AATGGACTTGAATCGTAGGT
CCL28-R	AAGGCTGAGGTAGGAGAAT
CD83-F	CGCAGCTCTCCTATGCAGTG
CD83-R	GTGTTTTGGATCGTCAGGGAATA
CXCL12-F	CACTGCCTATGTCCTCTTC
CXCL12-R	ACTGTTCTCCTGCTCCTT
FOXP3-F	CCCATCCCCAGGAGTCTTG
FOXP3-R	ACCATGACTAGGGGCACTGTA
GAPDH-F	AGGTCGGTGTGAACGGATTTG
GAPDH-R	TGTAGACCATGTAGTTGAGGTCA
GM-CSF-F	GGCCTTGGAAGCATGTAGAGG
GM-CSF-R	GGAGAACTCGTTAGAGACGACTT
HSV-1-ICP0-F	CCTCCTCCGCCTCTTCCTCTG
HSV-1-ICP0-R	GCGTCTTCCTGGCACACTTCC
HSV-1-UL41-F	GCGAACCTCTATCATACCAA
HSV-1-UL41-R	GCAGTGAATCGTCGGAAT
IFN-α-F	CTTCCTCAGACTCATAACCT
IFN-α-R	AGTCCTTCCTGTCCTTCA
IFN-β-F	GATGAACTCCACCAGCAGACAGTG

IFN-β-R	CACCATCCAGGCGTAGCTGTTG
IFN-γ-F	ATCAGGCCATCAGCAACAACA
IFN-γ-R	CGTCTCACCTCAAACTTGGCA
IKKβ-F	CTCCGAAGATACTTGAACCA
IKKβ-R	CGATGCGATGTCACTCAG
IL-12-F	TGGTTTGCCATCGTTTTGCTG
IL-12-R	ACAGGTGAGGTTCACTGTTTCT
IL-23-F	ATGCTGGATTGCAGAGCAGTA
IL-23-R	ACGGGGCACATTATTTTTAGTCT
IL-4-F	GTGAGCTCGTCTGTAGGGCT
IL-4-R	CCGCTTACCGATGAATCCAGG
IL-6-F	TAGTCCTTCCTACCCCAATTTCC
IL-6-R	TTGGTCCTTAGCCACTCCTTC
IRF4-F	TCCGACAGTGGTTGATCGAC
IRF4-R	CCTCACGATTGTAGTCCTGCTT
MHCI-F	CCTACCAGAGAATGATTGGCTG
MHCI-R	GCAACTCATGCAGGTTGGC
P38-F	AGACCGTTTCAGTCCATC
P38-R	CACCAGGTACACGTCATT
RORγt-F	GACCCACACCTCACAAATTGA
RORγt-R	AGTAGGCCACATTACACTGCT
Runx1-F	GCAGGCAACGATGAAAACTACT
Runx1-R	GCAACTTGTGGCGGATTTGTA
Runx3-F	CAGGTTCAACGACCTTCGATT
Runx3-R	GTGGTAGGTAGCCACTTGGG
STAT1-F	TCACAGTGGTTCGAGCTTCAG
STAT1-R	GCAAACGAGACATCATAGGCA
STAT3-F	CAATACCATTGACCTGCCGAT
STAT3-R	GAGCGACTCAAACTGCCCT

STAT6-F	CTCTGTGGGGCCTAATTTCCA
STAT6-R	CATCTGAACCGACCAGGAACT
T-bet-F	AGCAAGGACGGCGAATGTT
T-bet-R	GGGTGGACATATAAGCGGTTC
TGF-β-F	CTCCCGTGGCTTCTAGTGC
TGF-β-R	GCCTTAGTTTGGACAGGATCTG
TNF-α-F	GCCAACGGCATGGATCTCAA
TNF-α-R	TCTTGACGGCAGAGAGGAGG
ZAP70-F	CTACGTGCTGTCGTTGGTG
ZAP70-R	GTTACACGGCTTACGCAGGT
